# Association of perinatal factors with suspected developmental delay in urban children aged 1–36 months - a large-scale cross-sectional study in China

**DOI:** 10.1186/s12887-022-03819-9

**Published:** 2023-01-06

**Authors:** You Yang, Lei Shi, Xingming Jin, Shilu Tong

**Affiliations:** 1 Department of Developmental and Behavioral Pediatrics, Shanghai Children’s Medical Center, Shanghai Jiaotong University School of Medicine, 1678 Dongfang Road, Shanghai, 200127 People’s Republic of China; 2 Department of Pediatrics, Shanghai Fengxian District Hospital of Traditional Chinese Medicine, Shanghai, People’s Republic of China; 3 Department of Clinical Epidemiology and Biostatistics, Shanghai Children’s Medical Center, Shanghai Jiaotong University School of Medicine, 1678 Dongfang Road, Shanghai, 200127 People’s Republic of China; 4 School of Public Health, Institute of Environment and Population Health, Anhui Medical University, Hefei, People’s Republic of China; 5 Center for Global Health, School of Public Health, Nanjing Medical University, Nanjing, People’s Republic of China; 6 School of Public Health and Social Work, Queensland University of Technology, Brisbane, Australia

**Keywords:** Children, Perinatal factors, Developmental delay, Infants, Toddlers, Body mass index

## Abstract

**Background:**

Studies on perinatal risk factors and the developmental delay of children have been inconclusive and few studies have assessed the association between infants and toddlers’ body mass index (BMI) and developmental outcomes.

**Methods:**

We conducted a cross-sectional study of children aged 1—36 months who had a routine physical examination in the child health departments of hospitals from March 2018 to November 2021 in 16 provinces, 4 autonomous regions and 2 municipalities directly under the central government by using the Infant Toddler Growth Development Screening Test (ITGDST). Normal children were defined as those with scores ≥ mean – 2 standard deviations (SD), while children with developmental delay were those with scores < mean—2SD in terms of overall development, gross motor, fine motor and language development. Binary logistic regression was used to analyze the risk factors of gross motor, fine motor, language and overall neurodevelopment.

**Results:**

After removing some provinces with a small sample size and children with incomplete data, 178,235 children with 12 complete variables were included in the final analysis. The rate of overall developmental delay was 4.5%, while 12.5% of children had at least one developmental delay aspect. Boys, parity, advanced maternal age, multiple birth, cesarean section, neonatal injury, family heredity history, microcephaly, abnormal BMI at birth and at physical examination after controlling the confounding of other factors had a significant effect on development delay (overall neurodevelopment, gross motor, fine motor or language development). Per capita gross domestic product was a protective factor for the children’s neuropsychological development.

**Conclusions:**

This study reveals significant associations of perinatal factors and BMI with developmental delay in the Chinese children aged 1–36 months, which may be crucial for early intervention.

## Background

Child development can be affected by a combination of socioeconomic, environmental and nutritional factors during pregnancy and the early stage of life [1, 2]. Several studies have demonstrated the impact of nutrition on children’s cognition [3–5]. One study has shown that malnutrition was associated with increasing developmental deficits including suboptimal cognition, communication, and motor function in children [6]. On the other hand, children with severe obesity are more likely to have poor non-verbal intelligence quotient [7]. Other studies have documented the effect of sociodemographic variables on neuropsychological development, including child gender [8], ethnicity [9], economic situation [7].

In China, the National Survey on Physical Growth and Development of Children (NSPGDC) was conducted every 10 years in nine cities among children under 7 years. Although there were rapid positive secular trends in height and weight in both urban and suburban children from 1975 to 2005 [10], a recent NSPGDC conducted in 2015 displayed a new trend of slowing growth in urban children [11]. Both under- and over-nutrition in children are major global public health challenges [12, 13]. Since 2000, China has made remarkable progress in reducing child mortality, child malnutrition, and child at risk of poor neurodevelopment [14–16]. However, the gaps of those health indicators between developed and underdeveloped areas in China did not narrow as fast as the reduction of their national prevalence [14, 15, 17].

Furthermore, studies on perinatal risk factors and the developmental delay of children have been inconclusive [18]. In addition, few studies have compared the association of body mass index (BMI) with different developmental delays, even though early childhood nutrition is the foundation of neurodevelopment. In the present study, we aimed to determine whether perinatal and other risk factors were associated with children’s developmental delay through a large-scale cross-sectional study in Chinese cities.

## Methods

### Study population

**Fig. 1:**
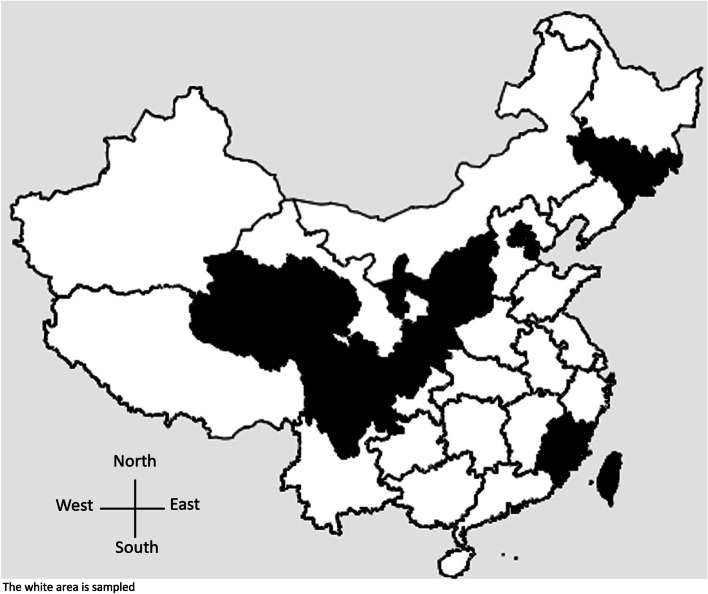
Regional distribution map of study population

In order to analyze the current situation and perinatal risk factors of developmental delay among children aged 1—36 months, children aged 1 to 36-months from the general population had a routine physical examination in the child health departments of hospitals from March 2018 to November 2021 in 16 provinces (Anhui, Gansu, Guangdong, Guizhou, Hainan, Hebei, Henan, Heilongjiang, Hubei, Hunan, Jiangsu, Jiangxi, Liaoning, Shandong, Yunnan, Zhejiang), 4 autonomous regions (Guangxi Zhuang Autonomous Region, Inner Mongolia Autonomous Region, Tibet Autonomous Region, Xinjiang Uygur Autonomous Region) and 2 municipalities (Shanghai and Chongqing) directly under the central government (Fig. 1). These regions took the lead in adopting better screening instrument for growth and development, since the existing Denver developmental screening test has not been updated for many years. During the physical examination, most parents or caregivers only provided information for assessing the health status of children, and some of the information was due to their concern on privacy. After removing some provinces with a small sample size (less than 1000) and children with incomplete data, 178,235 children with 12 complete variables were included in the final analysis.

### Case identification and grouping

The Infant Toddler Growth Development Screening Test (ITGDST, Shanghai Mengbaobao Health Technology Co., Ltd) can be used to screen for abnormal growth and development in children aged 1–36 months. The mean scores minus two standard deviations (SD) were used for the cut-off scores in terms of overall development, gross motor, fine motor and language development. Children with a score less than the mean score minus 2 SD were regarded as a developmental delay, while other children (i.e., a score equal to or greater than the mean score minus 2 SD) were considered as normal.

### Data collection

In this study, ITGDST was used for collecting children’s basic information and evaluating children’s physical and neuropsychological development. All testers (doctors or nurses) undertook unified on-site training and assessment. The test was conducted in a separate and quiet room with plenty of light. The room temperature was set at around 25 ℃. Children were awake and quiet. Parents and caregivers were asked to complete the neuropsychological evaluation item by item with the animation demonstration and the testers’ instruction. The ITGDST evaluation usually takes less than 10 min. The system also collected children’s information about the perinatal period, parents and environment and inheritance. Parents provided the following information about their children: date of birth, gender, birthweight, length, gestational weeks (< 37 weeks, 37–42 weeks, and ≥ 42 weeks), normal delivery (yes or no), maternal age ≥ 35 years (yes or no), neonatal injury (yes or no), multiple birth (yes or no), cesarean section (yes or no), family heredity history (yes or no). The per capita gross domestic product (GDP) of each region is included as a continuous variable.

Head circumference, height and length were measured by using the Full Function Physical Examination Instrument (Shanghai Beigao Medical Technology Co., Ltd.) during physical examination by trained testers. The instrument is automatically calibrated when it is turned on. The measurement usually takes less than 5 min. The physical development of infants and young children was evaluated by Z-score recommended by the World Health Organization (WHO). Normal head circumference (-2 ≤ Z-score ≤ 2), macrocephaly (Z-score > 2) and microcephaly (Z-score < -2) were defined according to head circumference for age Z-score by the standard of WHO. Body weight and length values were converted into BMI as weight per height squared (kg/m^2^). Normal children (-2 ≤ Z-score ≤ 2), children with malnutrition (Z-score < -2) and obesity (Z-score > 2) were determined according to BMI for age Z-score by the standard of WHO.

### Statistical analysis

Student t-tests, chi-squared tests, and logistic regression models were used to assess the associations of perinatal and other risk factors with the children’s developmental delay. Student t-test was used to assess the difference of Per capita GDP. Chi-squared tests were used to assess the differences of qualitative variables. The normal distribution test on the observation values of the quantitative data was made by using histogram and Quantile–Quantile plot. We used complete data and there was no imputation of data to replace missing observations. Binary logistic regression model was used to investigate the effect of relevant factors on developmental status of children (developmental delay and normal development). Adjusted odds ratios (ORs) with their 95% confidence intervals (CIs) were generated. A *p* value < 0.05 was set as the significant level (two tailed). All analyses were conducted using IBM SPSS version 22.0 (IBM Corp., Armonk, NY, USA).

## Results

### Description of the study population

The sample consisted of 178,235 children aged 1–36 months with complete data. The median age of children was 6.60 (1.08–36.99) months. More than half of the study population comprised males (53.9%). Most of the subjects were 1–12 months old, accounting for 68.4%. About 20% of children were aged 12–24 months. Most children (91.2%) came from 11 provinces including Anhui, Gansu, Henan, Heilongjiang, Hubei, Hunan, Jiangsu, Jiangxi, Shandong, Yunnan and Zhejiang. Birth weight and birth length were 3.39 (± 0.45) kg and 50.19 (± 1.45) cm, respectively. The malnutrition rate of children at birth was higher than that at physical examination (4.3% vs. 1.0%, *p* < 0.0001), while the rate of obesity at physical examination was higher than that at birth (6.5% vs. 2.6%, *p* < 0.0001). Table 1 shows the sample characteristics.

**Table 1:** Sociodemographic characteristics of the sample

Characteristics		Number (%) or mean (± SD)^a^
Gender	Female	82,095 (46.1)
	Male	96,140 (53.9)
Age	1–12 months	121,987(68.4)
	13–24 months	34,950 (19.6)
	25–36 months	21,298 (11.9)
Region	11 provinces	163,855(91.9)
	3 autonomous areas	10,780(6.0)
	1 municipality	3600(2.0)
Birth weight		3.39 ± 0.45^a^
Birth length		50.19 ± 1.45^a^
Nutritional status at birth	Normal	165,833 (93.0)
	Malnutrition	7700 (4.3)
	Obesity	4702 (2.6)
Head circumference at physical examination	Normal	168,544(94.5)
	Macrocephaly	7084 (4.0)
	Microcephaly	2607 (1.5)
Nutritional status at physical examination	Normal	164,779(92.5)
	Malnutrition	1823 (1.0)
	Obesity	11,633 (6.5)
Developmental delay	Overall neurodevelopmental delay	8068 (4.5)
	Gross motor delay	9014 (5.1)
	Fine motor delay	8786 (4.9)
	Language developmental delay	10,769 (6.0)

^a^ Means and standard deviations for continuous variables

### Assessment of potential risk factors for developmental delay

Table 2 examines the association of perinatal and other factors with the developmental delay. The results of the univariable analysis showed that sex, parity of more than three children, advanced maternal age, multiple birth, cesarean section, neonatal injury, family heredity history, microcephaly, abnormal BMI at birth and at physical examination were significantly associated with developmental delay.

**Table 2:** Comparison between normal and developmental delayed children with respect to perinatal and physical examination variables

	Overall neurodevelopment			Gross motor development			Fine motor development			Language development		
	Normal	Delayed	*P* value	Normal	Delayed	*P* value	Normal	Delayed	*P* value	Normal	Delayed	*P* value
	N(%)	N(%)		N(%)	N(%)		N(%)	N(%)		N(%)	N(%)	
Variables in terms of children												
Child—Sex												
Female	78,652(95.8)	3443(4.20)		77,976(95.0)	4119(5.0)		78,349(95.4)	3746(4.6)		77,773(94.7)	4322(5.3)	
Male	91,515(95.20)	4625(4.80)	< 0.001*	91,245(94.9)	4895(5.1)	0.476	91,100(94.8)	5040(5.2)	< 0.001*	89,693(93.3)	6447(6.7)	< 0.001*
Parity of more than three children												
No	169,686(95.5)	8029(4.5)		168,741(95.0)	8974(5.0)		168,971(95.1)	8744(4.9)		166,982(94.0)	10,733(6.0)	
Yes	481(92.5)	39(7.5)	0.001*	480(92.3)	40(7.7)	0.006*	478(91.9)	42(8.1)	0.001*	484(93.1)	36(6.9)	0.398
Multiple birth												
Singleton	168,824(95.5)	7978(4.5)		167,894(95.0)	8908(5.0)		168,107(95.1)	8695(4.9)		166,143(94.0)	10,659(6.0)	
Twins/multiple birth	1343(93.7)	90(6.3)	0.001*	1327(92.6)	106(7.4)	< 0.001*	1342(93.6)	91(6.4)	0.013*	1323(92.3)	110(7.7)	0.009*
Gestational weeks												
37–42 weeks	168,663(95.5)	7998(4.5)		167,719(94.9)	8942(5.1)		167,955(95.1)	8706(4.9)		166,002(94)	10,659(6)	
< 37 weeks	795(95.2)	40(4.8)	0.715	795(95.2)	40(4.8)	0.721	790(94.6)	45(5.4)	0.539	767(91.9)	68(8.1)	0.011*
≥ 42 weeks	709(95.9)	30(4.1)	0.542	707(95.7)	32(4.3)	0.366	704(95.3)	35(4.7)	0.810	697(94.3)	42(5.7)	0.690
Neonatal injury												
No	169,718(95.5)	8028(4.5)		168,778(95.0)	8968(5.0)		169,000(95.1)	8746(4.9)		167,008(94)	10,738(6)	
Yes	449(91.8)	40(8.2)	< 0.001*	443(90.6)	46(9.4)	< 0.001*	449(91.8)	40(8.2)	0.001*	458(93.7)	31(6.3)	0.782
Head circumference at physical examination												
Normal	160,926(95.5)	7618(4.5)		160,051(95)	8493(5)		160,277(95.1)	8267(4.9)		158,364(94.0)	10,180(6.0)	
Macrocephaly	6782(95.7)	302(4.3)	0.308	6723(94.9)	361(5.1)	0.83	6715(94.8)	369(5.2)	0.247	6674(94.2)	410(5.8)	0.382
Microcephaly	2459(94.3)	148(5.7)	0.005*	2447(93.9)	160(6.1)	0.011*	2457(94.2)	150(5.8)	0.047*	2428(93.1)	179(6.9)	0.079
BMI at birth												
Normal	158,521(95.6)	7312(4.4)		157,593(95)	8240(5)		157,845(95.2)	7988(4.8)		155,964(94.0)	9869(6.0)	
Malnutrition	7177(93.2)	523(6.8)	< 0.001*	7162(93)	538(7)	< 0.001*	7154(92.9)	546(7.1)	< 0.001*	7106(92.3)	594(7.7)	< 0.001*
Obesity	4469(95)	233(5)	0.073	4466(95)	236(5)	0.876	4450(94.6)	252(5.4)	0.087	4396(93.5)	306(6.5)	0.112
BMI at physical examination												
Normal	157,423(95.5)	7356(4.5)		156,565(95.0)	8214(5.0)		156,811(95.2)	7968(4.8)		154,885(94.0)	9894(6.0)	
Malnutrition	1697(93.1)	126(6.9)	< 0.001*	1649(90.5)	174(9.5)	< 0.001*	1696(93)	127(7)	< 0.001*	1701(93.3)	122(6.7)	0.219
Obesity	11,047(95.0)	586(5.0)	0.004*	11,007(94.6)	626(5.4)	0.058	10,942(94.1)	691(5.9)	< 0.001*	10,880(93.5)	753(6.5)	0.040*
Variables in terms of parents												
Advanced maternal age												
No	165,341(95.5)	7765(4.5)		164,375(95.0)	8731(5.0)		164,610(95.1)	8496(4.9)		162,683(94.0)	10,423(6.0)	
Yes	4826(94.1)	303(5.9)	< 0.001*	4846(94.5)	283(5.5)	0.127	4839(94.3)	290(5.7)	0.015*	4783(93.3)	346(6.7)	0.032*
Cesarean section												
No	98,392(95.6)	4578(4.4)		97,895(95.1)	5075(4.9)		97,959(95.1)	5011(4.9)		96,842(94.0)	6128(6.0)	
Yes	71,775(95.4)	3490(4.6)	0.055	71,326(94.8)	3939(5.2)	0.004*	71,490(95)	3775(5)	0.151	70,624(93.8)	4641(6.2)	0.060
Variables in terms of the environment and inheritance												
Per capita GDP	3.62 ± 0.96a	3.40 ± 0.82 a	< 0.001*	3.62 ± 0.96 a	3.51 ± 0.86 a	< 0.001*	3.63 ± 0.96 a	3.30 ± 0.81 a	< 0.001*	3.62 ± 0.96 a	3.39 ± 0.83 a	< 0.001*
Family heredity history												
No	169,631(95.5)	8032(4.5)		168,693(95.0)	8970(5.0)		168,928(95.1)	8735(4.9)		166,940(94.0)	10,723(6.0)	
Yes	536(93.7)	36(6.3)	0.042*	528(92.3)	44(7.7)	0.004*	521(91.1)	51(8.9)	< 0.001*	526(92)	46(8)	0.044*

Abbreviations: *GDP* Gross domestic product, *BMI* Body mass index

^*^Significant at 0.05

^a^ Means and standard deviations and 95% confidence intervals for continuous variables, per 10,000 Chinese yuan

Table 3 reveals the adjusted ORs for factors associated with the developmental delay. Boys, parity, advanced maternal age, multiple birth, cesarean section, neonatal injury, family heredity history, microcephaly, abnormal BMI at birth and at physical examination had a significant effect on developmental delay after controlling the potential confounding factors. Per capita GDP was protective factors for the children’s neuropsychological development.

**Table 3:** Factors associated with developmental delay according to multivariable logistic regression analysis

	Overall neurodevelopment		Gross motor development		Fine motor development		Language development	
	OR(95%CI)	P value	OR(95%CI)	*P* value	OR(95%CI)	*P* value	OR(95%CI)	*P* value
Variables in terms of children								
Child—Sex								
Female	1.00 (Reference)		1.00 (Reference)		1.00 (Reference)		1.00 (Reference)	
Male	1.15(1.1–1.21)	< 0.001*	1.01(0.97–1.05)	0.651	1.15(1.1–1.2)	< 0.001*	1.29(1.24–1.35)	< 0.001*
Parity of more than three children								
No	1.00 (Reference)		1.00 (Reference)		1.00 (Reference)		1.00 (Reference)	
Yes	1.56(1.12–2.16)	0.008*	1.48(1.07–2.04)	0.018*	1.47(1.07–2.02)	0.017*	1.03(0..74–1.45)	0.848
Multiple birth								
Singleton			1.00 (Reference)		1.00 (Reference)		1.00 (Reference)	
Twins/multiple birth	1.17(0.94–1.46)	0.161	1.3(1.06–1.59)	0.013*	1.08(0.87–1.35)	0.477	1.12(0.91–1.37)	0.277
Neonatal injury								
No	1.00 (Reference)		1.00 (Reference)		1.00 (Reference)		1.00 (Reference)	
Yes	1.49(1.07–2.06)	0.018*	1.69(1.24–2.29)	0.001*	1.21(0.87–1.68)	0.260	0.83(0.58–1.2)	0.322
Head circumference								
Normal	1.00 (Reference)		1.00 (Reference)		1.00 (Reference)		1.00 (Reference)	
Macrocephaly	0.94(0.84–1.06)	0.334	1.01(0.91–1.13)	0.793	1.07(0.96–1.19)	0.247	0.96(0.87–1.06)	0.414
Microcephaly	1.21(1.02–1.43)	0.026*	1.18(1.01–1.39)	0.040*	1.12(0.95–1.33)	0.175	1.1(0.94–1.28)	0.217
BMI at birth								
Normal	1.00 (Reference)		1.00 (Reference)		1.00 (Reference)		1.00 (Reference)	
Malnutrition	1.47(1.34–1.62)	< 0.001*	1.35(1.23–1.48)	< 0.001*	1.36(1.24–1.49)	< 0.001*	1.23(1.12–1.34)	< 0.001*
Obesity	1.13(0.99–1.29)	0.075	1(0.88–1.15)	0.971	1.12(0.99–1.28)	0.079	1.11(0.98–1.25)	0.095
BMI at physical examination								
Normal	1.00 (Reference)		1.00 (Reference)		1.00 (Reference)		1.00 (Reference)	
Malnutrition	1.36(1.13–1.64)	0.001*	1.83(1.56–2.15)	< 0.001*	1.23(1.02–1.48)	0.027*	0.98(0.81–1.18)	0.804
Obesity	1.16(1.06–1.26)	0.001*	1.1(1.01–1.19)	0.029*	1.28(1.18–1.39)	< 0.001*	1.09(1.01–1.18)	0.033*
Variables in terms of parents								
Advanced maternal age								
No	1.00 (Reference)		1.00 (Reference)		1.00 (Reference)		1.00 (Reference)	
Yes	1.29(1.14–1.45)	< 0.001*	1.1(0.97–1.25)	0.132	1.14(1.01–1.28)	0.040*	1.11(0.99–1.24)	0.067
Cesarean section								
No	1.00 (Reference)		1.00 (Reference)		1.00 (Reference)		1.00 (Reference)	
Yes	1.03(0.98–1.08)	0.250	1.06(1.01–1.11)	0.026*	1.02(0.98–1.07)	0.399	1.03(0.99–1.07)	0.214
Variables in terms of the environment and inheritance								
Per capita GDP	0.79(0.77–0.81)	< 0.001*	0.88(0.86–0.9)	< 0.001*	0.69(0.68–0.71)	< 0.001*	0.78(0.76–0.79)	< 0.001*
Family heredity history								
No	1.00 (Reference)		1.00 (Reference)		1.00 (Reference)		1.00 (Reference)	
Yes	1.47(1.04–2.06)	0.027*	1.58(1.16–2.15)	0.004*	2.05(1.53–2.74)	< 0.001*	1.44(1.06–1.95)	0.019*

Abbreviations: *OR* Odds ratio, *CI* Confidence interval, *GDP* Gross domestic product, *BMI* Body mass index

^*^Significant at 0.05

## Discussion

Adverse birth outcomes such as low birth weight and preterm have been reported to be associated with suboptimal developmental outcomes [19–22]. However, few large, population-based studies have assessed the association of perinatal factors and other variables at physical examination with developmental outcomes in newborns, infants, toddlers [23]. We assessed the association of pregnancy and neonatal factors and BMI at physical examination with overall neurodevelopment, gross-motor, fine-motor and language development. Our results show that the rate of overall developmental delay was 4.5%, while 12.5% of children had at least one developmental delay aspect, which is consistent with a previous study reporting that 5–17% of children suffered from developmental disabilities [8]. Preterm infants were more likely to have developmental delay. In this study, prematurity was a significant risk factor for language development in the univariate analysis, but in the multivariable logistic regression model, after taking into account the effects of confounding factors, it was no longer statistically significant. Our result is consistent with the report of Gurka et al. [24].

In the present study, boys had a higher rate of overall developmental delay, fine motor and language delay, which is consistent with previous studies [8, 25, 26]. According to the study by Whitehouse et al., a high level of testosterone in the male umbilical cord was a risk factor for speech developmental delay at the age of 1, 2 and 3 years old [27]. Research also suggests the effect of epigenetic mechanisms on sex differences in the human brain [28, 29]. In the study of Martínez-Nadal et al., cesarean delivery was associated with the risk of developmental delay [30], especially in the gross motor area, which is consistent with our study. Mehreen et al. also found the differences remained for gross-motor skills at the 12-month assessment between infants born by caesarean section and vaginally born [31]. However, in the study by de. Moura et al., cesarean section did not have a significant relationship with the developmental delay of children [26]. In the study of Kerstjens et al., cesarean section had a significant correlation with developmental delay in a univariate analysis but the significance disappeared after adjustment for confounding factors in multivariable analysis [32]. The cause of cesarean section and experimental design may account for different conclusions [33].

From early childhood through adolescence, higher family income tends to be associated with higher scores on assessments of language, memory, self-regulation, and social-emotional processing [34–37]. Early childhood poverty has been associated with differences in brain structure and function. The causal impact of a poverty reduction intervention on brain activity in the first year of life has been reported [38]. Such changes reflect neuroplasticity and environmental adaptation and display a pattern that has been associated with the development of subsequent cognitive skills [38]. According to the previous studies and our result, it suggests that economical advantage may be linked with differences in brain structure among children for their neurodevelopment.

In the current study, we found a significant association of mothers’ parity of more than three children with gross motor, fine motor and overall developmental delay, which is consistent with a recent study [39]. The mother’s parity of more than three children may be linked with socioeconomic status, which may limit adequate child care and nurturing. The findings suggest that parity was an independent risk factor for the children’s neurodevelopment. The association between advanced maternal age and neonatal outcomes remains controversial. In one study, advanced maternal age did not affect any short-term outcomes. However, at 2 years of corrected age, advanced maternal age was associated with a higher incidence of severe speech delay, even after controlling other confounding factors [40]. The statistically significant association between advanced maternal age and developmental delay (overall development and fine motor) was observed in our study in the multivariate (adjusted) model. The mechanism might be due to alterations in DNA methylation and changes in the expression of miRNAs regulating neuronal plasticity [41].

In the present study, the history of neonatal injury has a significant relationship with developmental delay. Infants with neonatal injury can have conditions like periventricular leukomalacia. Severe germinal matrix-intraventricular hemorrhage, and post-hemorrhagic hydrocephalus, which may directly affect developmental outcomes [42]. Also, neonatal birth injury also brings the risk of neurodevelopmental delay due to increased hospital stay [18]. In this study, family heredity history was associated with the risk of developmental delay. Family background has long been known to play an important role in influencing patterns of phenotypic expression in genetic syndromes and common diseases. The inheritance is complex because it involves multiple pairs of genes, as well as other environmental factors [43]. Many studies have suggested that shared molecular pathways could account for the multiple clinical signs that characterize neurodevelopmental disorders [44, 45]. The severity and variability of neurodevelopmental features is contingent upon family history of neuropsychiatric disease [46].

Microcephaly is a clinical finding and a crude but trusted assessment of intracranial brain volume. Microcephaly may develop at birth for developmental processes reducing in utero neuron generation or after birth for predominant dendritic or white matter diseases [47]. Some families showing autosomal dominant microcephaly have normal intelligence, psychometric evaluation of microcephalic children [48]. Children born with microcephaly associated with congenital Zika virus have a significant neurodevelopmental delay [49]. In our study, microcephaly was associated with the risk of gross motor and overall developmental delay. For its heterogeneous etiology, the family history of microcephaly needs further inquiry and increasingly genomic tests are available that allow an exact diagnosis.

In terms of multiple birth, twins are considered to be at an increased risk for neurodevelopmental impairments [50]. Triplet or higher-order births are associated with an increased risk of neurodevelopmental impairment [51, 52]. The area most at risk of delay is language. Twins had cognitive and neuropsychological outcomes that were otherwise comparable with singletons, but they had a slightly lower verbal intelligence quotient [53], which is consistent with our results.

Few studies have assessed the association between infants and toddlers’ BMI and developmental outcomes. In this study, abnormal BMI at birth and at physical examination was significantly associated with impaired gross motor, fine motor, language and overall development. This finding is consistent with the results of other studies. Previous studies showed that low birth weight, small-for-gestational-age or stunting newborns are associated with an increased risk of developmental delay in motor and behavioral evaluation [32, 54, 55]. Many children born with low BMI may have had chronic nutritional needs and oxygen during the fetal period. These chronic defects may alter the formation of neuronal connections and structure of the brain [32, 56]. It is estimated that approximately 25–50% of infants with low birth weight have brain abnormalities associated with cognitive, behavioral, attentional, and socialization impairment [56, 57].

Childhood obesity has also become a global concern. Obesity begins early in life and has been associated with impaired cognition [58]. Nutritional and syndromic obesity due to chromosomal or monogenic defects has attendant co-morbidities, which may include neurodevelopmental delays. Possible mechanisms include altered brain structure, leptin/insulin regulation, oxidative stress, cerebrovascular function, blood–brain barrier, inflammation, and decreased motor performance associated with a degraded musculoskeletal system [59]. Individualized nutrigenomic managements of obesity should be highlighted.

This study has three major strengths. First, it is the first large-scale cross-sectional study of development screening in China. Second, a large sample size enforces the precision of the study. Finally, all neuropsychological evaluation was conducted item by item with the animation demonstration and the doctors’ instruction to ensure the screening accuracy. This study is, however, also limited in several ways. First, children who participated in this study were not randomly selected so the potential for selection bias cannot be ruled out. Second, there may be greater subjectivity of self-reported information to assess perinatal and other risk factors. Additionally, the data on home environment, parental characteristics and individual-level economic factors were unavailable in the study. Third, as a cross-sectional study, the reliance on associations at a single time point make it inadequate for evaluating the causality between exposure and response variables. A cohort study of multiple time points is required for the association of perinatal factors with developmental delay.

## Conclusions

This study reveals significant associations of perinatal factors and BMI with developmental delay among children aged 1–36 months in China, which may be crucial for early intervention. The Infant Toddler Growth Development Screening Test appeared to be a useful instrument to screen for abnormal growth and development in young children.

## Data Availability

The datasets used and/or analysed during the current study are available from the corresponding author on reasonable request.
